# Modified Leak-Proof Puncture Technique for the Aspiration of Giant Ovarian Cysts by Instantly Mounting a Plastic Wrap and Gauze with Cyanoacrylates: A Retrospective Observational Study

**DOI:** 10.3389/fsurg.2022.948073

**Published:** 2022-07-05

**Authors:** Hiroshi Ishikawa, Makio Shozu

**Affiliations:** Department of Reproductive Medicine, Graduate School of Medicine, Chiba University, Chiba, Japan

**Keywords:** minimally invasive surgical procedures, ovarian cysts, cyanoacrylates, leak-proof puncture, minilaparotomy, plastic wrap

## Abstract

**Objective:**

We developed a leak-proof puncture technique for giant ovarian cysts by instantly mounting a plastic wrap to the cysts using cyanoacrylates and aspirating cyst fluid over the wrap. Here, we modified it by inserting a gauze between the wrap and cyst to strengthen the mounting. This study aimed to clarify the feasibility of the modified procedure.

**Method:**

A retrospective observational study was conducted in a single center. Surgical outcomes of 35 women who underwent the modified procedure from December 2013 to July 2020 were compared with those of 51 women who underwent the original procedure.

**Results:**

Mean long-axis diameters of the cysts were 233.1 mm and 229.8 mm in the modified and original procedures, respectively. The median of surgical time, blood loss, and aspirated fluid volume were 109 min, 50 ml, and 3,050 ml, in the modified procedure, all of which were not significantly different from those of the original procedure. One case of mounting disruption and two (5.7%) cases of intraperitoneal spillage of the cyst fluid were observed in the modified procedure, whereas four (7.8%) cases of mounting disruption and five (9.8%) cases of intraperitoneal spillage occurred in the original procedure. These events were caused by aspiration difficulty of the high viscosity fluid and/or multilocular cysts. Laparotomy conversion was observed in five (14.3%) cases in the modified procedure.

**Conclusion:**

Our modified procedure is feasible in select cases. The high viscosity of the cyst fluid and multilocular cyst may cause mounting disruption and intraperitoneal spillage of the cyst fluid.

## Introduction

The leak-proof puncture technique for the aspiration of cyst fluid in giant ovarian cysts is necessary for the removal of the cysts through small abdominal incisions and may prevent intraperitoneal spillage of cyst fluid during minimally invasive surgery. Several puncture techniques to aspirate the cyst contents without leakage during laparoscopic surgery have been reported (1–3). We had earlier reported a leak-proof puncture technique using cyanoacrylates and plastic wrap through mini laparotomy (4).

Our original technique consisted of instantly mounting a plastic wrap to the cyst using cyanoacrylates, followed by cyst fluid aspiration over this wrap. This technique is applicable to large and multiple cysts that exceed the umbilical height. However, the mounting between the wrap and the cyst was occasionally disrupted because of its fragility, resulting in the unexpected leakage of cyst fluid during aspiration. To reinforce the mounting between the wrap and the cyst, we modified the technique by inserting a gauze between the cyst and the wrap. Herein, we have reviewed a case series of the modified procedure and compared its surgical outcomes with those of the original procedure to clarify the feasibility and determine the appropriate recipients of the modified procedure.

## Materials and Methods

This is a retrospective observational study conducted in a single center. The study protocols for data analysis, including referring patient records, were approved by the Institutional Review Board at the Graduate School of Medicine, Chiba University (No. 2267). For this study, the opt-out method was applied to obtain consent for reviewing the patient records.

The study participants were women who underwent resection of giant ovarian cysts using the leak-proof puncture technique by instantly mounting a plastic wrap to the cysts using cyanoacrylates in the Chiba University Hospital. We have introduced the modified procedure in December 2013. Accordingly, we reviewed the medical records of 35 women who underwent the modified procedure in our facility from December 2013 to July 2020. To validate the modified procedure, we reviewed data of 51 women who underwent the original procedure for giant ovarian cyst fluid aspiration from January 2006 to September 2013.

The leak-proof puncture technique was applied to treat giant ovarian cysts that were determined to be benign tumors, including those which we cannot completely deny as borderline malignant tumors on preoperative imaging, mainly magnetic resonance imaging (MRI). Medical image interpretation specialists of our facility participated in a conference to determine the surgical indications relevant to the procedure.

**Figure 1:**
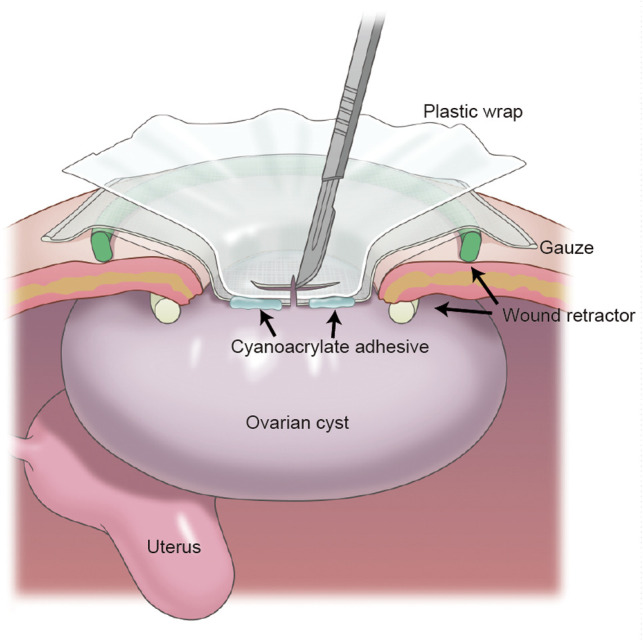
Illustration of the modified leak-proof puncture technique for the aspiration of giant ovarian cysts. This illustration shows a cross-section of the modified leak-proof puncture technique for fluid aspiration of giant ovarian cysts. A wound retractor is attached to the abdominal small incision, and a plastic wrap and gauze are mounted to the cyst instantly using cyanoacrylate adhesive. After the mounting process, the cyst wall is cut and the cyst fluid is aspirated over the wrap.

**Figure 2:**
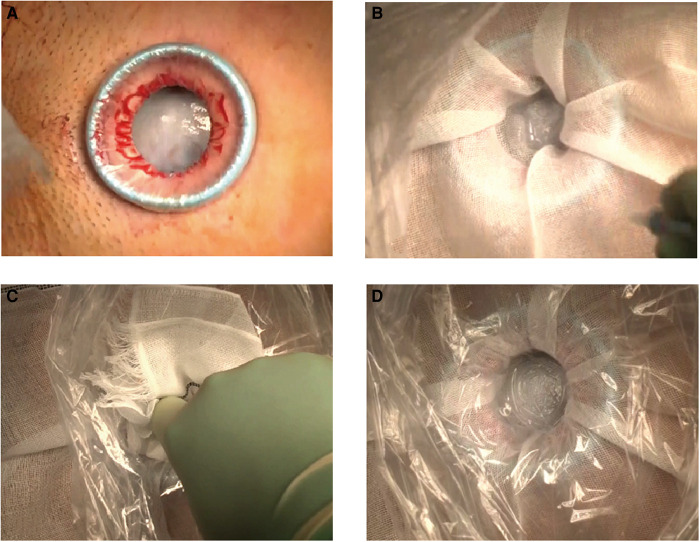
Instant mounting of plastic wrap and gauze to the giant ovarian cyst. (**A**) Completion of the attachment of a wound retractor to the 3-cm lower abdominal transverse incision. (**B**) Condition immediately after applying cyanoacrylates on the cyst through the gauze. (**C**) Mounting the plastic wrap and gauze on the cyst. (**D**) Completion of the instant mounting to the cyst wall. The color of the adhesive has changed from transparent to white upon completion of cyanoacrylate polymerization.

**Figure 3:**
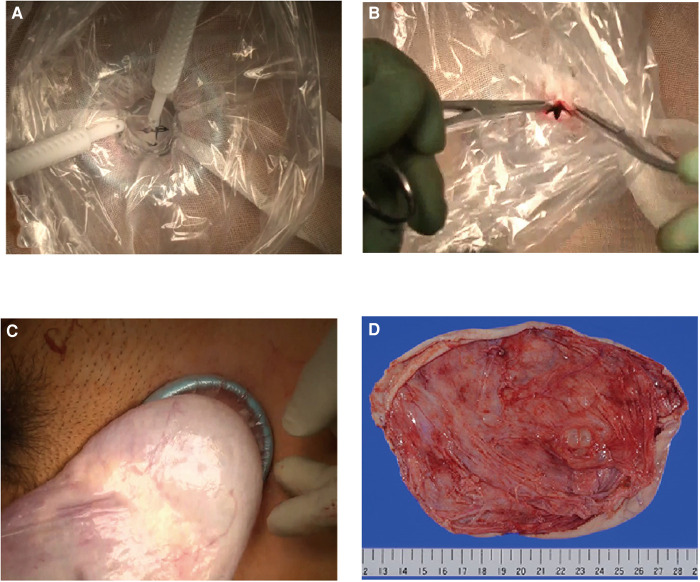
Aspiration of the cyst fluid and cystectomy of the giant ovarian cyst. (**A**) Aspirating cyst fluid over the wrap. The cyst fluid of the serous cystadenoma flows from the puncture hole. (**B**) After cyst fluid aspiration, the mounting is pinched off with forceps to avoid intraperitoneal spillage of the cyst fluid. (**C**) The cyst is brought outside the body. (**D**) Resected giant ovarian cyst. The longitudinal diameter of the cyst is 205 mm. The pathological diagnosis was serous cystadenoma.

An illustration of the modified procedure, consisting of the instant mounting of the plastic wrap and gauze to the cyst using cyanoacrylate adhesive and cyst fluid aspiration, is presented in Figure 1. First, we made a 3–5 cm transverse or vertical incision in the lower abdomen and attached a disposable retractor (Alexis® Wound Retractor; Applied Medical, Rancho Santa Margarita, CA, USA or other similar retractors) to visualize the cyst surface (Figure 2A). Then, we removed moisture from the cyst surface, placed a sterile gauze on the cyst, and applied cyanoacrylates (Aron Alpha®; Dai-ichi Sankyo, Tokyo, Japan) in a 2–4 cm diameter circle over the gauze (Figure 2B). Subsequently, we pressed a plastic wrap onto the gauze surface for 3 min (Figure 2C). The color of the adhesive changed from transparent to white following the completion of polymerization (Figure 2D). Subsequently, we punctured the cyst through the wrap with a sharp-pointed knife (Figure 1). The cyst fluid, with low viscosity, exuded from the cyst and was aspirated *via* the puncture hole (Figure 3A). Direct insertion of aspiration tubes into the puncture hole should be avoided to prevent the mounting from tearing. For multilocular cysts, initially, we aspirated the fluid in the largest cyst using the modified technique, punctured the adjacent cyst septum, and aspirated the cyst fluid. In cases where the adjacent cyst wall was not visible through the puncture hole, we pulled the cyst wall using forceps and extended the wall incision under direct vision. After the maximum possible aspiration of the cyst fluid, we lifted the cyst wall with the forceps (Figure 3B), placed it outside the body (Figure 3C), and performed cyst resection (cystectomy) or oophorectomy (Figure 3D). We also presented a representative video (Supplementary Material).

We reviewed patients' age, body mass index (BMI), histories of laparotomy and/or laparoscopy as patients' characteristics and preoperative estimation of the long-axis diameter of the cysts that was measured by MRI. We also reviewed the surgical time, blood loss volume, and aspirated cyst fluid as surgical outcomes. Additionally, we analyzed the reasons for mounting disruption and/or intraperitoneal spillage of the cyst fluid. We also analyzed the reasons for conversion to laparotomy during the modified procedure. If the surgeon has little experience of the modified procedure, an experienced physician participated in the surgery as the first assistant.

JMP 14.0 (SAS Institute, Inc., Cary, NC) was used for statistical analyses. The comparison of continuous and categorical variables between women who underwent the modified procedure and those who underwent the original procedure was performed using the Student t-test and Pearson's chi-square test, respectively. Statistical significance was defined as p < 0.05.

## Results

Patient characteristics and surgical outcomes are presented in Table 1. Patients' age, BMI, and long-axis diameter of the cysts were not significantly different between women who underwent the modified and original procedures. The mean long-axis diameters of the cysts were 233.1 mm and 229.8 mm in the modified and original procedure, respectively. The median surgical time, blood loss volume, and aspirated cyst fluid in the modified procedure were 109 min, 50 ml, and 3,050 ml, respectively, whereas those in the original procedure were 118 min, 150 ml, and 2,800 ml, respectively. These parameters were not significantly different between the modified and original procedures except for blood loss volumea. Histopathology revealed that most of the ovarian cysts in the modified procedure were mucinous cyst adenomas.

**Table 1:** Patient characteristics and surgical outcomes.

	Modified procedure (*n* = 35)	Original procedure (*n* = 51)	*p*-value
Patient characteristics			
Age (year)^a^	50.3 ± 21.5	46.6 ± 23.0	0.4500
BMI (kg/m^2^)^a^	22.6 ± 3.7	22.2 ± 4.0	0.6622
Long axis diameter of the cysts (mm)^a^,^c^	233.1 ± 61.9	229.8 ± 69.7	0.8246
Surgical outcomes			
Surgical time (min)^b^	109 (56–238)	118 (50–296)	0.1471
Volume of blood loss (ml)^b^	50 (5–590)	150 (10–5,335)	0.0387
Volume of aspirated cyst fluid (ml)^b^	3,050 (800–13,800)	2,800 (400–14,000)	0.6115

^a^ *Data are presented as mean ± standard deviation.*

^b^ *Data are presented as median (range).*

^c^ *Diameter calculated by preoperative imaging evaluation.*

Mounting disruption was observed in only one case in the modified procedure, whereas it was observed in four cases in the original procedure. Intraperitoneal spillage of the cyst fluid did not occur in the modified procedure because the fluid was highly viscous. Conversely, spillage was observed in all cases in the original procedure. The disruption and subsequent spillage occurred at the aspiration of multilocular cysts. Intraperitoneal spillage of the cyst fluid that was not accompanied by mounting disruption during surgery was observed in two cases in the modified procedure and two cases in the original procedure. The spillage occurred during the aspiration of multilocular cysts and/or the aspiration of the highly viscous fluid. Disruption of the cyst wall has occurred instead of the mounting disruption in these cases.

Conversion to laparotomy was observed in five cases in the modified procedure. Reasons for the conversion were as follows: difficulty of aspiration over the wrap because of the highly viscous cyst fluid (two cases), difficulty in picking up the entire cyst after cyst fluid aspiration due to broad and firm adhesions between the cyst and rectosigmoid colon caused by prior hysterectomy (two cases), and difficulty of mounting a plastic wrap and gauze due to the retroperitoneal location of the cyst (one case). No recurrent cases associated with intraperitoneal spillage of cyst fluid were observed.

## Discussion

Intraperitoneal spillage of the cyst fluid of ovarian cystic tumors should be avoided during resection, even if they are not suspected of malignancy, preoperatively. When intraperitoneal spillage of the cyst fluid occurs, peritoneal lavage is necessary. Before the introduction of the modified procedure, we had occasionally experienced intraperitoneal spillage of the cyst fluid because of the mounting disruption during the aspiration procedure. Mounting disruption may be due to the fragility of the adhesive surface. The gelatinous, highly viscous cyst fluid is challenging to aspirate over the wrap; hence, the fluid from inside the cyst is directly aspirated, leading to a strong tension over the mounting that causes its disruption. We had experienced four cases of mounting disruption out of 51 cases before the modification, and this disruption has shown an evident reduction with the modified technique (1 of 35 cases), though this difference was not significantly significant.

Although similar leak-proof puncture techniques using Dermabond and Dermabond plus (Johnson &Johnson Inc., NJ, USA) and BioGlue (CryoLife, GA, USA) for instant mounting of a plastic bag, vinyl membrane, and surgical glove to ovarian cysts have been reported (5–7), to the best of our knowledge, our modification to secure the mounting has not yet been reported. Similar to the original procedure, the modified procedure is safe, quick, and easy to perform, without requiring specialized training. In addition, it can be applied to giant ovarian cysts where the upper border is over the umbilicus. Laparoscopic surgery for the resection of huge ovarian cysts has also been reported; however, preventive methods for intraperitoneal spillage during the suction of the cyst fluid have not been mentioned in most of them. Our modification can also be applied during laparoscopic-assisted surgery.

Inadequate intraperitoneal observation throughout the small incision is a disadvantage of the modified technique at mini laparotomy, as with the original technique. Intraperitoneal adhesions between the tumor and other organs may be unidentifiable. Laparoscopy-assisted surgery could resolve this problem. Appropriate reduction of the cyst volume will enable the safe insertion of laparoscopic trocars into the abdominal wall, after pneumoperitoneum is achieved. The wound retractor, in our case series, was covered with a surgical glove and the cap closed to avoid air leakage during laparoscopy. Other authors have reported that a laparoscopic cap was put on to create and maintain pneumoperitoneum at the laparoscopy (8). The combination of laparoscopic observation and subsequent release of the adhesion may be useful in avoiding laparotomy conversion in cases of adhesion between the cyst and pelvic walls or other intraperitoneal organs.

A prior history of hysterectomy is associated with intraperitoneal adhesion between the ovarian tumor and intraperitoneal organs, including the pelvic peritoneum, gastrointestinal tract, and omentum (9). The remaining adnexa are occasionally fixed to the pelvic side wall during hysterectomy; in such cases, placing the entire ovarian cyst outside the body was observed to be difficult even after complete aspiration of the cyst contents. Therefore, careful attention must be paid to patients with prior history of hysterectomy before the application of the modified procedure.

Based on the instructions, the Aron alpha ethyl-2-cyanoacrylate used in this study, can be used below skin level and also for vessel adhesion. It has been used for soft tissue bonding in dermatologic surgeries (10) and surgical adhesion to blood vessels in vascular surgeries (11). No toxicity issues with the glue have been reported so far. Furthermore, we developed our modified procedure to reduce the risks of glue spillage inside the abdominal cavity. The gauze inserted between the plastic wrap and the cyst wall helps to trap all the glue. From this point of view, our modified procedure is much safer than the original procedure.

Cost-effectiveness including operative time in the modified procedure should be discussed. Compared with the original procedure, the modified procedure only adds a sterile gauze. It takes several seconds to insert the gauze between the plastic wrap and the cyst wall, and thus the operative time in the modified procedure may be delayed by several seconds. On the other hand, the enforcement of the bonding makes it easy to pick up the cyst outside the body. This may shorten the subsequent resection of the cyst. From this point of view, the modified procedure is also cost-effective.

This study has several limitations. First, the sample size is relatively small. Therefore, we could not detect any significance in the frequency of mounting disruption, intraperitoneal spillage of the cyst fluid, and conversion to laparotomy between the modified procedure and the original procedure. Second, we chose women who underwent the original procedure for the control. The surgeons who performed the two procedures were different, which might affect the surgical outcomes. Third, surgeons' experience in performing the procedure is a potential bias for the surgical outcomes. Finally, surgical outcomes included not only the leak-proof puncture procedures but also the subsequent cystectomy or resection of the cysts. Surgical time may extend, and blood loss volume may increase in cases of laparotomy conversion.

This procedure is important for decreasing the risk of ovarian cancer upstaging. Although we did not apply the modified procedure to apparent malignant ovarian cystic tumors in preoperative imaging diagnosis, we experienced several cases that showed malignancy in postoperative pathological diagnosis. Fortunately, intraperitoneal spillage of the cyst fluid did not occur in these cases; nevertheless, making an effort to avoid intraperitoneal spillage during the procedures was necessary.

In conclusion, we have modified our leak-proof puncture technique for fluid aspiration in giant ovarian cysts by inserting a gauze between the plastic wrap mounting and cyst. This modification reinforces the mounting and may prevent its disruption. This technique is safe, quick, and easy to perform without cyst fluid leakage. Additionally, it does not require pneumoperitoneum; therefore, it can be applied to patients in which long-term pneumoperitoneum may be avoided, for example, pregnant women. It can also be applied in laparoscopic-assisted surgery.

## Data Availability

The raw data supporting the conclusions of this article will be made available by the authors, without undue reservation.
